# Antimicrobial Active Bioplastics Using Triangular Silver Nanoplate Integrated Polycaprolactone and Polylactic Acid Films

**DOI:** 10.3390/ma14051132

**Published:** 2021-02-28

**Authors:** Eduardo Lanzagorta Garcia, Olivia A. Attallah, Marija Mojicevic, Declan M Devine, Margaret Brennan Fournet

**Affiliations:** Materials Research Institute, Athlone Institute of Technology, Athlone N37 HD68, Ireland; oadly@ait.ie (O.A.A.); mmojicevic@ait.ie (M.M.); ddevine@ait.ie (D.M.D.); mfournet@ait.ie (M.B.F.)

**Keywords:** polycaprolactone, polylactic acid, antibacterial, triangular silver nanoplates, composite films

## Abstract

An innovative antimicrobial technology for plastic surfaces is presented. We report the synthesis and scale-up of triangular silver nanoplates (TSNPs) and their integration into polycaprolactone (PCL) and polylactic acid (PLA) polymers through a solvent-casting technique. The TSNPs have a high geometric aspect ratio and strong local surface plasmon resonance (LSPR) response, which provides an effective tool for monitoring their integrity during processing and integration with the biodegradable plastics. An aqueous-based seed-mediated chemical method was used to synthesize the TSNPs, and characterisation was carried out using TEM and UV (Ultraviolet)-VIS (Visible) spectroscopy to measure LSPR profiles. The UV-VIS spectra of silver seeds and TSNPs exhibited characteristic peaks at 395 and 600 nm respectively. Synthesized TSNPs were coated with thiol-terminated polyethylene glycol (SH-PEG) and transferred into chloroform in order to effect compatibility with PCL and PLA. TSNP/PCL and TSNP/PLA composite films were prepared by solvent casting. The morphological structure, thermal, mechanical, and antimicrobial properties of the TSNP-incorporated composite films were evaluated. Results showed the TSNP-treated films had a rougher surface than the bare films. Insignificant changes in the thermal properties of TSNP-treated films compared to bare ones were also observed, which indicated the thermal stability of the composite films. The tensile strength and antimicrobial properties of the composite films were increased after TSNP incorporation. TSNP/PCL and TSNP/PLA films exhibited improved antimicrobial activity against *Escherichia coli* and *Staphylococcus aureus* with antimicrobial effect (AE) values ranging between 0.10 and 0.35. The obtained results and demonstrated TSNP production scalability validate the TSNP treated PCL and PLA films as a composite material with desirable antimicrobial effect for wide-ranging surface applications.

## 1. Introduction

Surfaces, in particular common synthetic surfaces, are a frequent source of contamination and/or transmission of microbiological organisms and a critical aspect of disease and infection containment. This can be especially problematic at sites such as healthcare facilities, food industries, and public environments, among others, where pathogens can easily spread and lead to disease acquisition and transmission. Furthermore, antibiotic resistance in microorganisms is rising globally and severely compromising our ability to prevent and treat common infectious diseases [1].

The development of new antibiotic drugs is a long and investment-intensive process, with costs averaging USD 800 million and durations of 10 years or more required [2,3]. Additionally, there is no guarantee that a given drug will be legally approved for use or will generate sufficient profits, making it unattractive for pharmaceutical companies, hence there is a notable decrease in the release of approved antibiotics during the last decades. Thus, to address the growing problem of antimicrobial resistance, there is an urgent need for the development of novel and innovative alternative approaches. These new technologies must enable a reduced dependence on antibiotic drugs and deliver reduced risks of infection. As a result, researchers are focusing on new strategies such as including novel molecules and using informatics and nanotechnology for the development of novel anti-infection technologies.

Progress in nanoscience has provided a range of advanced functional nanomaterials that are paving the way to improved solutions for human well-being in several different fields. The applications of functional nanomaterials vary from energy to electronics to sensors, environmental, and drug delivery [4]. Metallic nanoparticles (NPs) such as copper oxide (CuO), titanium dioxide (TiO_2_), gold (Au), and iron oxide (Fe_2_O_3_) NPs have demonstrated significant antimicrobial potential [5]. Nevertheless, silver (Ag) remains one of the most attractive metals for the synthesis of NPs, because of the wide applications derived from its unique properties. Attributes like Ag’s malleability, conductivity, optical profile, and antimicrobial properties underpin its value and convenience for several functionalities and human requirements, through ancient and modern times [6,7].

Despite the numerous advantages and applications for Ag NPs, environmental risks are also a concern as the number of products using them increases rapidly. This will inevitably increase human and environmental exposure. Although the effects that nanomaterials could have on the environment are not fully understood yet, there is concern that some NPs could accumulate in target organs of the respiratory, cardiovascular, reproductive, urinary, and central nervous systems in exposed species [8]. Therefore, further studies are required to determine the extent of environmental risk, and to implement preventive measures accordingly. Humans are generally exposed to Ag from several sources, including jewelry, functional textiles, coins, tableware, deodorants, catheters, antibacterial treatments, coatings in refrigerators, etc. [9]. Several factors influence the cytotoxic level of Ag NPs, including size/shape of the nanoparticle, concentration, dose, surface chemistry and coatings on the NPs, the environment in which they interact and reside, and agglomeration [10]. Hence, more information is still required to correctly assess the hazards of chronic exposure to nano-silver and its ions, both for humans and the environment. For biomedical applications, it was found that the optimum particle size of Ag NPs to exert acceptable antimicrobial activity is in the range of 15–50 nm [11].

Triangular silver nanoplates (TSNPs) and other non-spherical Ag nanostructures with sharp geometries have been postulated to possess highly controllable optical properties related to strong electric-field enhancement that are especially pronounced in Ag nanostructures with tips [12]. TSNPs have been reported to exhibit higher antimicrobial activity, attributed to a rate of Ag^+^ ion release five times larger, compared to spherical NPs. As Ag^+^ ion release is caused by oxidation, the increased ion release of TSNPs is a consequence of their sharp edges and corners, which make them more susceptible to oxidation [13,14,15,16,17]. It is also recognised that larger surface-to-volume ratio possessed by smaller NPs compared to larger ones allows smaller particles to release more Ag^+^ ions. These results support the suitability of TSNPs to deliver antimicrobial activity for devices and surfaces.

Integration of Ag NPs into biodegradable polymers as polycaprolactone (PCL) and polylactic acid (PLA) for composite-film fabrication has been explored, and significant improvement was noticed in the antimicrobial properties of the Ag NP-treated composite films [18,19,20,21]. Nevertheless, one of the major challenges for the incorporation of Ag NPs in polymers is the nonhomogeneous distribution of NPs in the polymer matrix. Such insufficient dispersion, caused by NP agglomeration, leads to the deterioration of the optical and mechanical properties of the polymer nanocomposites [22]. Generally, the synthesis of Ag NPs is usually performed in aqueous systems due to the poor solubility of Ag ion salts in organic solvents, and also due to the increased environmental favourability of aqueous-based chemistries. Aqueous conditions, however, strongly hinder the compatibility of Ag NPs with hydrophobic polymer matrices during blending [23]. Therefore, to increase such compatibility strategies including surface modification, ultrasonic oscillations, and mechanical alloying have been proposed [24].

In the specific case of TSNPs, only a limited number of reports for their incorporation as fillers in composites to improve composites’ antimicrobial properties are reported. The functionalization of TSNPs on bulk glass surfaces designed to impart an antibacterial effect based on the release of Ag^+^ has been studied in [4]. A report by Vukoje et al. (2014) discussed the incorporation of TSNPs in cotton fabrics pretreated with chitosan for improvement of the antimicrobial properties of the fabrics. Results showed that the TSNPs were able to increase the antibacterial activity against Gram-negative *E. coli*, Gram-positive *S. aureus,* and yeast *Candida albicans* with a bactericidal percentage of 99% [25]. Furthermore, despite the diversity of TSNPs’ applications and their incorporation into polymers for the improvement of their electrical and thermal conductivity [26,27,28,29,30], there is a lack of information about TSNP integration into bioplastics for antimicrobial purposes. Combining the urgent need to develop innovative antimicrobial technologies that combine environmental sustainability and plastic-circularity principles, here we present antimicrobial biodegradable plastics based on TSNPs incorporated into PCL and PLA films. Furthermore, the polyester nature of PCL and PLA plastics is showing promise for closed-loop circularity developments, making these polymers, upon the incorporation of antimicrobial TSNPs, highly lucrative as biodegradable antimicrobial plastics. This study describes a solvent-casting technique for the preparation of TSNP-integrated polymer films with improved antimicrobial properties using PCL and PLA as model polymers for film processing. The morphological structure, thermal behavior, and mechanical properties of the fabricated films were characterized in detail. The antibacterial activity of TSNP-treated films was evaluated against *Escherichia coli* and *Staphylococcus aureus* strains. A new scaled-up TSNP production methodology is demonstrated, validating the industrial-scale applicability to this antimicrobial technology. This demonstration of antimicrobial active TSNP-integrated PCL and PLA bioplastics, which combine cost-effectivity with industrial scalability, is antibiotic-free and highly relevant, particularly given the current Covid-19 pandemic.

## 2. Materials and Methods

### 2.1. Materials

The HPLC-grade water (34877-2.5L), sodium citrate tribasic (C8532-100G) [TSC], poly(sodium 4-styrenesulfate) (434574-5G) [PSSS], sodium borohydride (213462-25G) [NaBH_4_], silver nitrate (204390-10G) [AgNO_3_] and L-ascorbic acid (A92902-25G) [AA] were obtained from Sigma-Aldrich Ireland Ltd. (Arklow, Ireland). An FRX pump from Syrris (Royston, UK) was used for the addition of AgNO_3_. O-[2-(3-mercaptopropionylamino)ethyl]-O’-methylpolyethylene glycol (11124-1G-F) The [SH-PEG] was obtained from Sigma-Aldrich Ireland Ltd. The PCL CAPA 6250 [MW 25,000] was obtained from Perstorp (Malmö, Sweden). The PLA Ingeo 4044D was obtained from NatureWorks LLC (Minnetonka, MN, USA).

### 2.2. Synthesis of Triangular Silver Nanoplates (TSNPs)

The TSNP synthesis was performed using a seed-mediated approach, adapted from [31]. This method involved two steps: seed production and TSNP growth. For the seed production, 5 mL of TSC (2.5 mM), 0.25 mL of PSSS (500 mg/L), and 0.3 mL of NaBH_4_ (10mM) were mixed, followed by the addition of 5 mL of AgNO_3_ (0.5 mM) at the rate of 2 mL/min with constant stirring. TSNP growth was carried out in 4 mL of distilled water (DW), 0.075 mL of AA (10mM), and 0.35 mL of seed solution (25.56 ppm of Ag), with the addition of 3 mL of AgNO_3_ (0.5 mM) at a rate of 1 mL/min. Finally, 0.5 mL of TSC (25 mM) was added. The finished reaction resulted in a final Ag concentration of 21.34 ppm.

### 2.3. Scale-Up of TSNP Production

Scaling up the TSNP synthesis was achieved by increasing all the reactants’ volumes from the adapted method proportionally to reach a final volume of 200 mL. An Ag seed volume of 20 mL was prepared by mixing 8.53 mL DW, 0.95 mL TSC (25 mM), 0.47 mL PSSS (500 mg/mL), and 0.57 mL NaHB_4_ (10 mM). A volume of 9.48 mL of AgNO_3_ (0.5 mM) was added at a rate of 3.79 mL/min. After 4 hours, the preparation of the seeds was complete and 8.75 mL of seed solution (25.56 ppm) was mixed with 100 mL of DW and 1.87 mL of AA (10 mM). 75 mL of AgNO_3_ (0.5 mM) was added at a rate of 25 mL/min, followed by 12.5 mL of TSC (25 mM) after the reaction was finished.

### 2.4. Transfer of TSNP to Chloroform

The prepared TSNP solution was concentrated with thermal evaporation at 40 °C for 8 days in an oven, to obtain an estimated final concentration of 43 ppm. The transfer of TSNP into chloroform was performed based on a previously reported method [23] with slight modification. Briefly, the TSNPs were coated with thiol-terminated polyethylene glycol (SH-PEG) where an amount of 4 mg of SH-PEG per millilitre of TSNP solution was stirred for a couple of minutes. Afterwards, the treated TSNPs were mixed with chloroform in a 1:1 proportion and centrifuged at 16.162 rcf for 45 minutes, and the supernatant was discarded.

### 2.5. TSNP Integration into PCL and PLA Biopolymers via Solvent-Casting Technique

The solvent-casting procedure for the PCL and PLA included the dissolution of 800 mg of polymer pellets in 15 mL of chloroform at room temperature, then 5 mL of TSNPs in chloroform were mixed with polymer solutions separately, and each mixture was poured in a Petri dish and dried overnight in an airtight oven at 40 °C. The treated films had a final concentration of 0.026 wt % of TSNPs. Bare films of PCL and PLA were prepared using the same procedure without the addition of TSNPs for further analysis comparisons.

### 2.6. Antimicrobial Activity 

The antimicrobial activity of casted films was evaluated against *Escherichia coli* ATCC 9001 (LGC Standards, Middlesex, UK) and *Staphylococcus aureus* ATCC 25923 (LGC Standards, Middlesex, UK) in Luria-Bertani broth (10 g/L tryptone, 10 g/L NaCl, 5 g/L yeast extract, pH 7.2) using adapted standard broth microdilution assay for bacteria that grow aerobically, as recommended by the CLSI (Clinical and Laboratory Standards Institute 2015). Prepared films (1 cm^2^) were used as samples, with bare films as a negative control and tested microorganisms as a positive control. Growth of the respective test organisms (10^5^ CFU/mL) after 24 h at 37 °C was measured as optical density (OD) at 600 nm using a Biotek Synergy HT Microplate Reader (Biotek Instruments GmbH, Bad Friedrichshall, Germany). Serial dilution of cultivated broth was plated and incubated (30 °C, 24 h), and the number of colonies (CFU/mL) developed on the bare and treated films was calculated.

### 2.7. Instrumentation 

The equipment used for the characterization of the TSNPs and prepared films are discussed in detail in Supplementary Materials.

## 3. Results and Discussion

### 3.1. TSNP Synthesis

The TSNP synthesis was performed using a seed-mediated approach adapted from [31]. The method involved two steps; seed production and TSNP growth. NaBH_4_ was used for the reduction of AgNO_3_ and seed formation. The chemical reaction occurred as follows [32]:

AgNO_3_ + NaBH_4_ → Ag + 1/2H_2_ + 1/2B_2_H_6_ + NaNO_3_

TSC and PSSS were used as stabilizers for seed production, and both have been reported to aid the formation of nanoplates. It is postulated that citrate ions and PSSS (being a charged polymer) bind to the surface of the {111} facet of seed particles, and promote the growth of the NPs into a triangular plate shape [31,33].

During the growth step, the spherical Ag seeds reacted with new Ag^+^ ions in the presence of AA and citrate ions, forming the TSNP as follows [34]:

(Spherical Ag NPs) + Ag^+^ + C_6_H_8_O_6_ + C_6_H_5_Na_3_O_7_ → (TSNP)

**Figure 1:**
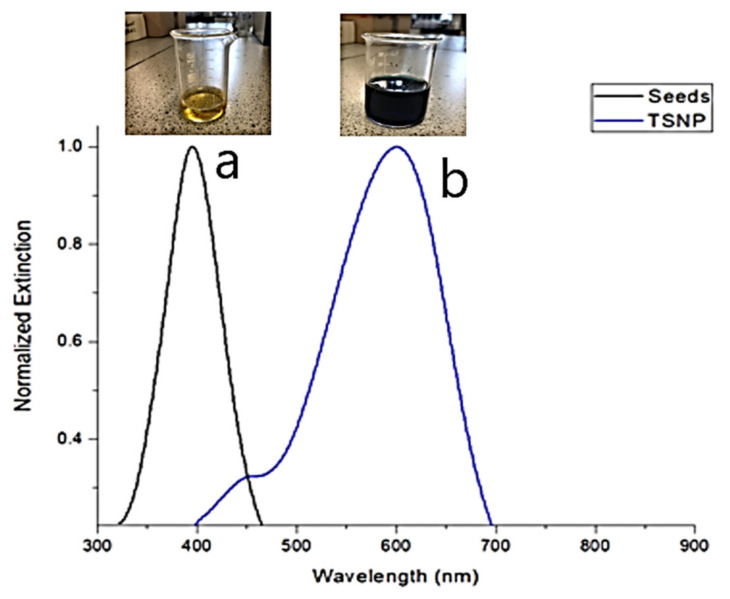
UV-VIS spectrum of (**a**) Ag seeds and (**b**) TSNPs.

It was found that upon the addition of AgNO_3_, the colour changed from yellow to shades of orange, then shades of red and purple, and finally a dark blue colour of TSNPs was obtained. The normalized UV-VIS spectrum of the seeds and the TSNPs prepared using 350 µL of seeds are shown in Figure 1. The spectrum of the seeds had a localized surface plasmon resonance (LSPR) peak at 395 nm and a full width at half maximum (FWHM) of 79 nm (435–356 nm). The TSNP curve had a peak at 601 nm and an FWHM of 150 nm (663–513 nm). These results are in accordance with the results obtained by the original method, indicating the successful production of TSNPs [31].

The remarkably strong local surface plasmon resonance (LSPR) of these TSNPs has previously been reported in [35]. The LSPR of these TSNPs provides a highly effective tool for monitoring the processing of the TSNPs, providing a means to directly follow the geometric evolution of the TSNPs, interactions at their surfaces, and compatibility with matrices in which they are embedded [36,37]. The LSPR response is hence available to provide a direct readout of the preservation of the TSNP integrity through the processing steps involved in the preparation of TSNP antimicrobial biodegradable plastic films.

**Figure 2:**
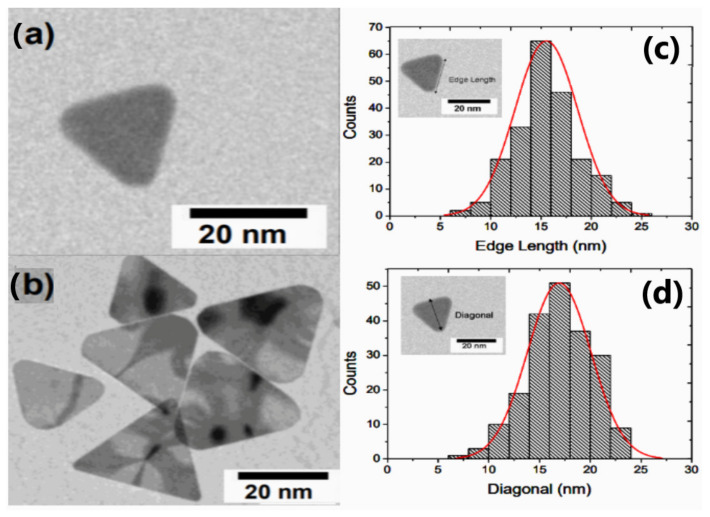
TEM images of (**a**) single TSNP, (**b**) multiple TSNP, (**c**) Edge length and (**d**) diagonal size distribution of TSNP.

As shown in Figure 2, transmission electron microscope (TEM) analysis confirmed the presence of triangular plates, with defined sharp corners having a mean edge length of 15 ± 4 nm and a mean diagonal of 17 ± 6 nm. While in the case of equilateral triangles, to which the TSNP can be approximated, the edge length was greater than the diagonal length, which was also true for the TSNPs, given the relatively high error in the measurement of the mean diagonal lengths.

The scaling up of the TSNP production was performed to evaluate the efficiency of the synthesis technique in the production of large volumes of TSNPs. As shown in Figure S1, the LSPR peak wavelength of the scaled-up TSNP batch was 584, with an FWHM of 112 nm (635–523 nm). An insignificant blue shift in the peak and a small reduction in FWHM was observed in comparison to that of smaller prepared volumes of TSNP.

**Figure 3:**
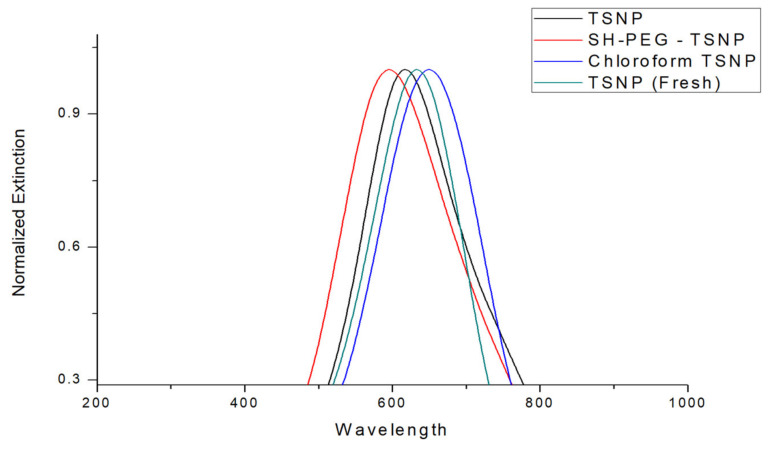
UV-VIS spectra of nonconcentrated TSNPs, concentrated TSNPs, SH-PEG-coated TSNPs, and TSNPs after transfer to chloroform.

A concentrated solution of TSNPs (43 ppm) was then obtained via thermal evaporation, as previously described. Figure 3 shows the UV-VISs spectrum of the TSNPs (21.34 ppm) before evaporation, with an LSPR peak wavelength of 633 nm suffering a small blue shift to 617 nm after thermal evaporation. The FWHM also exhibited a change, from 153 nm (706–553 nm) to 179 nm (723–544 nm) after evaporation. These differences between both LSPR spectral profiles were indicative of a slight broadening in the homogeneity of the TSPNs, with the minor blue shift and consistent spectral profile confirming that the triangular shape of the TSNP was preserved [35]. The scale-up process, therefore, did not significantly affect the geometric profile of the TSNPs, and hence was not an inhibitory process for the antimicrobial performance of the TSNPs.

In the current study, a solvent-casting technique was selected for demonstration of TSNP-incorporated biodegradable plastic film fabrication. One of the advantages of this technique is that it does not involve high temperatures, which can result in the fast oxidation of TSNPs. Nevertheless, the dissolution of all the film’s components in a single solvent is an essential requirement of the solvent-casting technique. The selected model biodegradable polymers (PCL and PLA) for the film production are hydrophobic polymers that have very poor solubility in water. TSNPs were therefore transferred from the aqueous phase to an organic phase. Chloroform was chosen as the organic solvent due to its high solubility for the model polymers, and SH-PEG was used to assist the transfer of TSNPs from the aqueous solution to chloroform [38]. UV-VIS spectra of the TSNPs before and after coating with SH-PEG, and after the transfer in chloroform, are presented in Figure 3. LSPR peak wavelengths were 596 nm for SH-PEG-coated TSNPs and 650 nm after transfer to chloroform, which was indicative of increased local refractive index at the TSNP surfaces due to the accumulation of SH-PEG. In comparison to the LSPR of concentrated TSNP, a blue shift of 21 nm occurred after coating the concentrated TSNP with SH-PEG, indicating that these TSNPs were, to a minor degree, more susceptible to oxidation following the concentration process. A subsequent red shift of 54 nm occurred for the concentrated SH-PEG-coated TSNPs upon transferring them to chloroform, indicating the preserved integrity of the TSNP geometries under these conditions. The FWHM was shifted to 197 nm (514–711 nm) for SH-PEG-coated and 168 nm (565–733 nm) for the chloroform solution. Noticeably, the most drastic shift in the LSPR and FWHM was observed during the transfer step. After transferring to chloroform, and contrary to the other shifts, a red shift occurred. Such a result can be attributed to the higher refractive index of chloroform (1.44) than water (1.33), which led to the production of red shifts in the LSPR of the TSNPs [39,40,41].

### 3.2. TSNP-Treated Polymer Films

**Figure 4:**
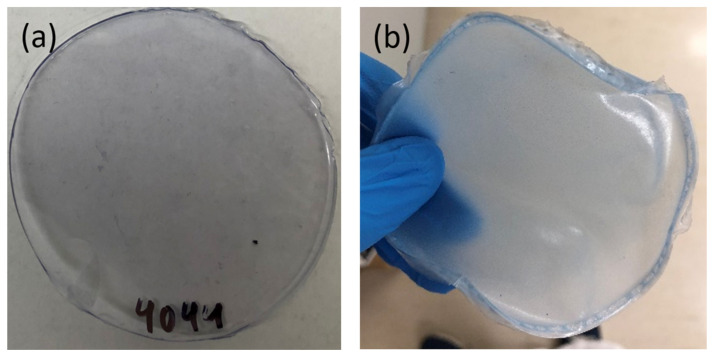
Photographic picture of (**a**) TSNP/PLA and (**b**) TSNP/PCL films.

Solvent casting was evaluated as a film-processing technique. Two model biodegradable polymers, PCL and PLA, were selected for the fabrication of TSNP-integrated composite films. Chloroform was used to dissolve the polymers and as suspension media for the SH-PEG-coated TSNPs. This resulted in better protection for the TSNPs from the air after solvent evaporation, allowing the films to maintain the blue colour of the TSNPs as shown in Figure 4. As mentioned before, the blue colour was an observable feature directly related to the LSPR properties of the TSNPs, serving as a straightforward indicator of the geometric integrity, as drastic changes in the geometry, would also lead to observable change or loss of colour. A similar example has been previously reported, in which blue TSNP-integrated cotton fabrics changed to yellow after autoclave sterilization, showing that agglomeration or transformation of the nanoplates into nanodiscs occurred [25].

#### 3.2.1. Scanning Electron Microscopy

**Figure 5:**
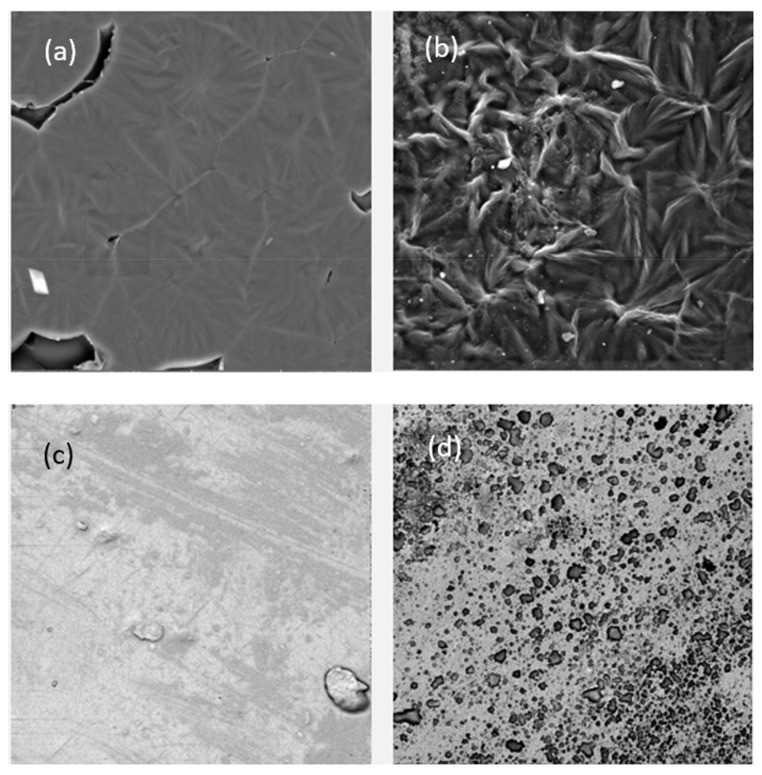
SEM images of: (**a**) PCL, (**b**) TSNP/PCL, (**c**) PLA, and (**d**) TSNP/PLA films (1000× magnification).

A Scanning lectron microscope (SEM) analysis was performed for the investigation of TSNP distribution in PCL and PLA films. The morphology of all the films is shown in Figure 5. The illustrative SEM images of the pure PCL and PLA films presented a good compact structure, with a smooth appearance. The images of TSNP-treated PCL (Figure 5b) and TSNP-treated PLA (Figure 5d) films showed rougher surfaces. On the whole, the TSNP had some tendency of aggregation in the PCL and PLA films. Such behaviour can be attributed to the high surface energy that TSNPs possess, which is affected by changes in the size of the NPs and environmental factors such as pH and ionic strength [42].

#### 3.2.2. Differential Scanning Calorimetry (DSC)

**Figure 6:**
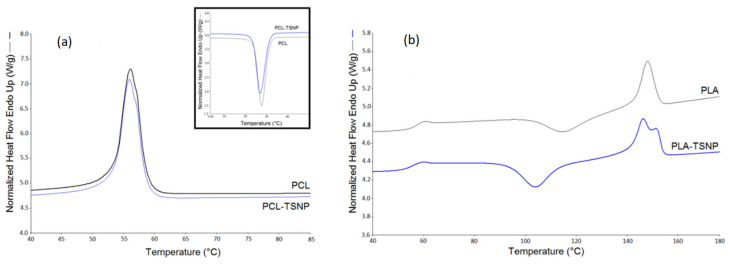
DSC curves for (**a**) bare and treated PCL films and (**b**) bare and treated PLA films.

The typical DSC curves of the bare and TSNP-treated PCL and PLA films are shown in Figure 6a,b, respectively. The glass transition (Tg), cold crystallization peak (Tc), melting process (Tm), and degree of crystallinity (Xc) data are summarized in Table 1. Noticeably, TSNPs had a limited effect on the thermal transitions of the treated polymers. For instance, compared to the bare PCL film, the addition of TSNPs did not affect the Tm (56 ℃) and Tc (27 ℃) of the PCL. Additionally, in treated PLA films, the values of Tg and Tm did not vary significantly from the bare PLA film, for which a change by 1–2 °C was only observed. This can be attributed to the rearrangements of the PCL and PLA chains promoted by the TSNPs, which resulted in the lowering of Tm by 1–2 °C [43]. These results were in accordance with previous reports in which the addition of Ag NPs did not result in obvious changes in the Tm values of PCL films [44]. Another study by Mróz et al. also found that nano-silver did not significantly affect the thermal transitions of treated PLA films [45].

Moreover, as listed in Table 1, the addition of the TSNPs led to an increase in the degree of crystallinity in PLA films of up to 27%, but no significant effect was observed in PCL films (Xc = 47%). In the case of treated PLA films, the resultant increase in the percentage of Xc can be explained by the phenomenon of heterogeneous nucleation. A similar result was obtained by Chu et al., where the Xc of PLA was increased as a result of addition of Ag NPs [46].

#### 3.2.3. Thermogravimetric Analysis (TGA)

**Figure 7:**
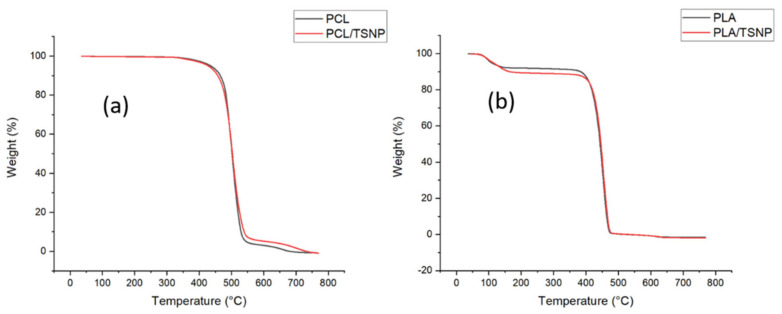
TGA curves of (**a**) bare and TSNP-treated PCL films, and (**b**) bare and TSNP-treated PLA films.

A TGA analysis was carried out to study the thermal stability of the prepared films. The TGA curves of the bare and TSNP-treated PCL films are shown in Figure 7a, while Figure 7b demonstrates the TGA curves of the bare and TSNP-treated PLA films. From the thermogravimetric curves, it can be observed that the bare and TSNP-treated films had a relatively good thermal stability, since all the maximum mass losses occurred between 400 and 500 °C. As shown in Figure 7a, the onset degradation temperature (Tonset) of pure PCL was approximately 439 °C, and the degradation was complete at about 550 °C. In PLA films, the Tonset was 380 °C, and complete degradation was observed at 475 °C. Both the Tonset and the completed degradation temperature of the TSNP-treated PLA and PCL films were not significantly different when compared with their pure films. Therefore, we can conclude that the addition of TSNPs obviously maintained the thermal stability of the PCL and PLA films. These results were in accordance with previously published studies in which no significant difference in thermal stability was observed upon the addition of nano-silver-coated chitosan to PLA films [47,48]. Similarly, the addition of different silver concentrations did not change the thermogravimetric behaviour of PLA samples after 7 days in composting conditions, as reported by [47]. On the other hand, Cerkez et al. (2017) and Leonés et al. (2017) concluded that PCL films and PCL electrospun fibers did not show a significant difference in thermal decomposition when treated with Ag NPs [49,50].

#### 3.2.4. Mechanical Properties

**Figure 8:**
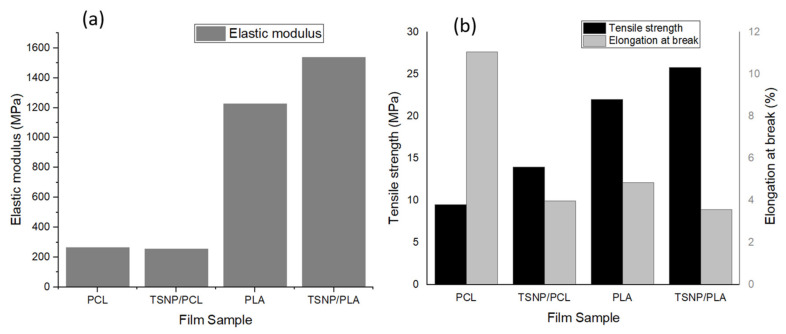
(**a**) Elastic modulus, (**b**) Tensile strength and Elongation at break of treated and bare PCL and PLA films

Tensile strength (TS), elastic modulus (EM), and elongation at break (ε) of the fabricated films were calculated as illustrated in Figure 8 The pure films of PCL and PLA exhibited a lower tensile strength (TS = 13.97 MPa for PCL and 21.99 MPa for PLA) and a higher percentage of elongation at break (ε = 11.07% for PCL and 4.84% for PLA) values compared to the TSNP-treated films. This was in accordance with Augustine et al. (2016), who reported that electrospun PCL membranes either maintained TS and EM values or exhibited an increase in those parameters at a higher percentage of added Ag NPs [51]. For PLA films, similar results were obtained by Ali et al. (2014), as they observed an increase in TS after the addition of Ag NPs to PLA [52]. Similarly, an increase in TS was also reported by Szymanska-Chargot et al. (2020) after fabricating a PLA composite containing Ag NPs and nanocellulose from carrot pomace [53]. In the current study, the increase of TS of PCL and PLA films after being mixed with the TSNPs can be attributed to the high compatibility between TSNPs and the model polymers. However, the TSNP-treated PCL films suffered a significant decrease in the elongation at break percentage, resulting in the formation of a very brittle film. Such a result might be due to TSNP stacking (as observed in SEM images), which reduced the mobility of the polymer chains. A similar outcome was also reported by Abdelaziz et al. (2020) as a result of adding hydroxyapatite nanoparticles to PCL composites [54].

Concerning the EM of the fabricated films, it was found that the incorporation of TSNPs led to an increase in the EM of the PLA film due to the high stiffness of the TSNPs as fillers compared to pure PLA. An increase in the EM as a result of Ag NP addition was also reported by Liu et al. (2017) and Szymanska-Chargot et al. (2020) [53,55]. Alternatively, there was no significant difference between the bare and TSNP-treated PCL films. A similar trend was also observed by Augustine et al. (2016), in which nanocomposites containing a small concentration of Ag NPs did not show a significant change in the elastic modulus of PCL composites [51].

### 3.3. Antimicrobial Activity

Dispersion of nanoparticles inside a polymer matrix can influence the mechanical and barrier properties, but also it can change its antimicrobial nature. As previously mentioned, TSNPs are proven to possess considerable antimicrobial activities, and as such the incorporation of TSNP in PCL and PLA films can improve the composites’ antibacterial characteristics. In our present study, both Gram-positive (*S. aureus* ATCC 25923) and Gram-negative (*E. coli* ATCC 9001) bacteria were used to assess the antimicrobial properties of TSNP-treated PCL and PLA composite films with respect to bare films. These bacterial strains were chosen as representatives of common harmful microorganisms occurring in various biomedical related products [56,57]. The antimicrobial effects (AE) of bare and TSNP-treated PCL and PLA composite films were then calculated using the following formula [4].

AE = log NC − log NE

where NC is the number of CFU/mL developed on the bare films, and NE is the number of CFU/mL counted after exposure to TSNP-treated films.

As shown in Table 2, all TSNP-treated films exerted an acceptable inhibitory effect against the tested Gram-positive and Gram-negative bacterial strains. Such antimicrobial activity was attributed to the TSNPs incorporated in the composite films since the bare PCL and PLA films showed no antibacterial activity. Additionally, solvent-casted TSNP/PLA films demonstrated higher AE values than TSNP/PCL films against both *S. aureus* and *E. coli,* which can be attributed to the easiness of Ag^+^ ions to be released from the surface of the PLA rather than the PCL matrix. Such results were in accordance with Ahi et al. (2019), who demonstrated that PLA films treated with a microbicidal compound such as propolis had a higher antimicrobial activity than PCL films did against *S. aureus* [58].

It is also worth mentioning that the TSNP-treated films showed increased antibacterial activity against Gram-negative bacteria than towards Gram-positive ones. This was due to the scarce thickness of the cell wall in the Gram-negative bacteria, making them more susceptible to the action of Ag^+^ ions released by the TSNPs in the treated films [59,60].

## 4. Conclusions

In this study, a solvent-casting technique for the incorporation of in-house fabricated TSNPs in PCL and PLA polymers was developed. The effect of TSNPs on the morphological structure, thermal properties, mechanical properties, and antimicrobial activity of PCL and PLA films was investigated. An SEM analysis showed the cross-section of the TSNP-treated films had a rougher surface than the bare films. The DSC curves confirmed that TSNPs had no effect on Tc and Tm values, but had an enhancing effect on the Xc percentage, especially for PLA. With the addition of TSNPs, TGA results showed maintenance in the thermal stability of the treated films. We found that with the incorporation of TSNPs in the model polymers, the composite films had a higher TS and lower elongation of break (ε). The EM of PLA films was also improved upon the addition of TSNPs, while no significant effect was observed on the EM of treated PCL films. Concerning the antimicrobial activity, the treated PCL and PLA films showed an enhanced growth inhibition for both *E. coli* and *S. aureus* strains, which was attributed to the bacterial inhibition ability of the TSNPs.

In conclusion, the obtained results clearly showed that these PCL and PLA films with incorporated TSNPs have considerable potential as bioplastics, with acceptable antimicrobial activity for surface and biomedical applications. Future work will constitute the optimization of TSNP-treated films to maximize their antimicrobial activity. In addition, a comparative study will be carried out to demonstrate the advantage of TSNPs over other morphologies of Ag NPs with respect to the antimicrobial activity of treated polymer films.

## Figures and Tables

**Table 1:** Thermal characteristics of the pure and TSNP-treated films.

Sample	Tg (°C)	Tm (°C)	Tc (°C)	Xc (%)
PCL	-	56.06	27.75	47.9
TSNP/PCL	-	55.92	27.36	47.6
PLA	56.7	148.1	114.86	21.5
TSNP/PLA	55.1	146.25	104	27.4

**Table 2:** Antimicrobial effect (AE) values * for TSNP-treated films.

Bacterial Strain	TSNP/PCL Films	TSNP/PLA Films
*E. coli* ATCC 9001	0.10	0.35
*S. aureus* ATCC 25923	0.11	0.28

* Values were obtained as the average of three measurements. AE = log N_C_ − log N_E._

## Data Availability

All data generated or analysed during this study are included in the article (and its Supplementary Information Files).

## Supplementary Materials

The following are available online at https://www.mdpi.com/1996-1944/14/5/1132/s1, Instrumentation, Figure S1. UV-VISs spectrum of Scaled-Up TSNPs (300 mL).
