# Supplementary material for: Antimicrobial Active Bioplastics Using Triangular Silver Nanoplate Integrated Polycaprolactone and Polylactic Acid Films
Source: Materials (Basel). 2021 Feb 28;14(5):1132. doi: 10.3390/ma14051132 (PMC7957712; doi:10.3390/ma14051132)
Supplement: Supplementary file 1 [file materials-14-01132-s001.pdf]

materials

Supplementary Materials

Antimicrobial Active Bioplastics Using Triangular Silver Nanoplate Integrated Polycaprolactone and Polylactic Acid Films

Eduardo Lanzagorta Garcia *, Olivia A. Attallah, Marija Mojicevic, Declan M Devine and Margaret Brennan Fournet

Materials Research Institute, Athlone Institute of Technology, Athlone N37 HD68, Ireland; oadly@ait.ie (O.A.A.); mmojicevic@ait.ie (M.M.); ddevine@ait.ie (D.MD.); mfournet@ait.ie (M.B.F.)

* Correspondence: e.lgarcia@research.ait.ie; Tel.: +353-083-352-7449

Instrumentation

Absorbance spectra of colloidal suspensions of silver seeds and TSNP were recorded using a Biotek Synergy HT Microplate Reader (Biotek Instruments GmbH, Germany) with Gen5 Microplate Reader Software from Biotek Instruments in the 300–900 nm range.

TEM images were obtained on colloidal solutions of TSNP diluted five times with distilled water and observed with a Jeol 2100 instrument (Jeol Ltd., Tokyo, Japan) using 64 Formvar coated copper grids.

SEM images were obtained using Mira XMU SEM (Tescan™, Brno, Czech Republic) in back scattered electron mode for surface analysis. The accelerating voltages utilized were 10 kV for PCL and 5 kV for PLA films. Prior to analysis, test samples were placed on an aluminum stub, and the samples were sputtered with a gold using Baltec SCD 005 for 110 s at 0.1 mbar vacuum before testing.

The thermal behaviour of the films was evaluated by a DSC Perkin Elmer 4000 (Perkin Elmer Washington, MA, USA) with Pyris Software (Version 13.3.1) under an inert nitrogen stream. About 10 mg of specimen was sealed in an aluminum pan and the DSC scans were recorded while heating from 10–200 °C at a heating rate of 10 °C/min, and then cooled to 10 °C. Thermal properties such as the glass transition temperature (Tg), melting temperature (Tm) and cold crystallization temperature (Tc) were obtained from the second heating scan. In addition, the percentage of crystallinity (Xc) was calculated according to the following Equation (1):

$$Xc\ (\%) = (\Delta Hm / W\Delta Hm_0) \times 100 \quad (1)$$

where ΔHm (J/g) is the heat of fusion of the sample. ΔHm_0 is the heat of fusion for completely crystalline PLA (93.7 J/g) [1] and PCL (136 J/g) [2] and W (g) is the weight fraction of PCL and PLA in the samples.

Thermal stability of the films was determined using a thermogravimetric analyzer Pyris TGA 1 (Perkin Elmer Washington, MA, USA) with software Pyris 1. The film samples were taken in a standard aluminum pan and heated from 30 to 600 °C at the rate of 10 °C/min under a nitrogen flow of 50 mL/min.

Mechanical properties of the films were tested using a 20 N tensometer Lloyd TA1 (Zwick Roell, Barcelona, Spain) with software Nexygen Plus version 3.0. All the samples were cut into 5 × 70 mm^2 pieces, and the tensile speed was 50 mm/min at room temperature according to ASTM D638. An average of six test values were taken for each sample.

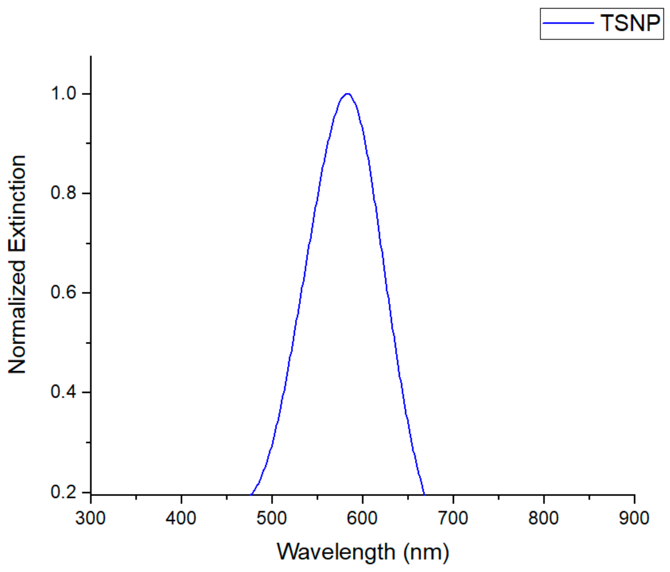

Figure S1. UV-Vis Spectrum of Scaled Up TSNP (300 mL).

References

1. Li, W.; Zhang, C.; Chi, H.; Li, L.; Lan, T.; Han, P.; Chen, H.; Qin, Y. Development of antimicrobial packaging film made from poly(lactic acid) incorporating titanium dioxide and silver nanoparticles. *Molecules* **2017**, *22*, doi:10.3390/molecules22071170.
2. Simão, J.A.; Bellani, C.F.; Branciforti, M.C. Thermal properties and crystallinity of PCL/PBSA/cellulose nanocrystals grafted with PCL chains. *J. Appl. Polym. Sci.* **2017**, *134*, doi:10.1002/app.44493.
